# Reconsidering the Levesque framework: a social work perspective for healthcare professionals

**DOI:** 10.3399/BJGPO.2022.0055

**Published:** 2022-07-13

**Authors:** Daisuke Nishioka

**Affiliations:** 1 Department of Medical Statistics, Research & Development Center, Osaka Medical and Pharmaceutical University, Osaka, Japan; 2 Department of Social Epidemiology, Graduate School of Medicine and School of Public Health, Kyoto University, Kyoto, Japan; 3 Miyama Rinken-Center Clinic, Kyoto, Japan

**Keywords:** Primary healthcare, General practice, Social work, Social determinants of health, Implicit bias

## Potential barriers to access primary care

Social backgrounds affect people’s health and health behaviour.^1^ Factors such as gender, marital status, race and/or ethnicity, nationality, income, education, job status, or social relationships are known as social determinants of health. Recently, healthcare professionals have been required to consider patients’ social determinants and to address issues to improve the care of patients.^2^

**Figure 1.**
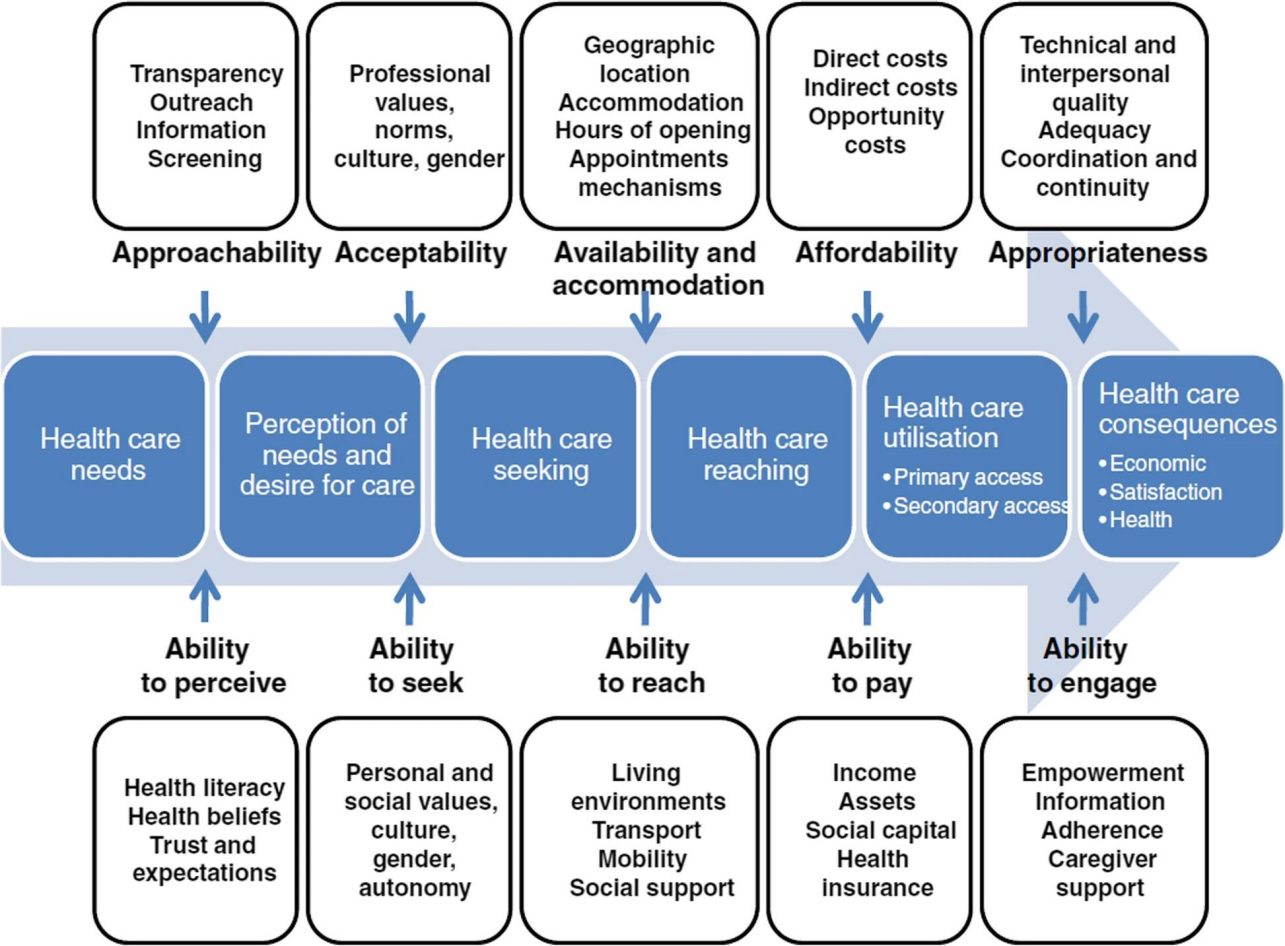
The conceptual diagram of the barriers and abilities required to achieve the benefits of primary care. Source: Levesque *et al* ^3^

These social determinants of health can also prevent patients’ access to primary care. According to Levesque *et al*, there are multiple barriers that people need to overcome to access primary care.^3^ The conceptual diagram which Levesque and colleagues previously summarised shows that the barriers include: ‘Approachability‘, ‘Acceptability‘, ‘Availability and accommodation‘, ’Affordability’, and ’Appropriateness’. To overcome the barriers, patients need to demonstrate their capabilities, which were summarised as ‘Ability to perceive‘, ‘Ability to seek‘, ‘Ability to reach‘, ‘Ability to pay‘, and ‘Ability to engage‘. Both barriers and capabilities are shown in Figure 1.

## Patients in front of us have the strength to overcome the barriers

The conceptual diagram can also be considered from the perspective of social work: we can regard the patient before us, who has accessed primary care and received care, as having the *strength* to demonstrate their capabilities and overcome the barriers to healthcare access. This perspective, known as a ‘strength model‘, is well known and utilised in the field of social work.^4^

However, healthcare professionals sometimes stigmatise socially vulnerable patients in clinical settings, being prejudiced against those patients capable of reaching primary care because of an unconscious implicit bias that is embedded in our daily practice.^5^ Once a patient is stigmatised by healthcare professionals, their power to overcome the barriers reduces due to discouragement, resulting in the state of ’powerlessness‘. Once the person became powerless due to stigma, they are being effectively excluded from the society.^6^ Consequently, the patients will be unable to access to primary care. This state is known as ’social withdrawal‘ and represents one of the coping skills of patients employ to avoid being stigmatised repeatedly.^7^ Such withdrawal may mean that the physician no longer sees the patient, leading to increased marginalisation.

If we learn from the social work perspective, we may be able to prevent this unfavourable consequence of implicit bias in healthcare professionals. Biestek‘s basic principles of social work include *individualisation, purposeful expression of feelings, controlled emotional involvement, acceptance, non-judgmental attitude, client self-determination, and confidentiality*.^8^ As we have seen, patients who could attain access to primary care had the strength to demonstrate their capabilities to overcome multiple barriers. We, as healthcare professionals, do not have the authority to judge an individual patient’s background. It is important to accept the process that has led the person to seek medical care support. Healthcare professionals have been reported to be one of the sources of patients’ stigma.^9^ Because stigma is a known health risk^10^ that can arise from healthcare professionals’ prejudice embedded in everyday life, it is important for healthcare professionals to be aware of their own implicit biases.^5^ Approaches utilised in social work, introduced above, may reduce the barriers faced by patients needing primary healthcare access, and may strengthen their capabilities. In recognising these concepts and their applicability to primary care, healthcare professionals may be able to encounter, with ‘fresh eyes’, a marginalised patient who had been prevented access to primary care due to their social determinants.
